# Fanconi Anemia Repair Pathway Dysfunction, a Potential Therapeutic Target in Lung Cancer

**DOI:** 10.3389/fonc.2014.00368

**Published:** 2014-12-19

**Authors:** Wenrui Duan, Li Gao, Brittany Aguila, Arjun Kalvala, Gregory A. Otterson, Miguel A. Villalona-Calero

**Affiliations:** ^1^Comprehensive Cancer Center, The Ohio State University College of Medicine and Public Health, Columbus, OH, USA; ^2^Division of Medical Oncology, Department of Internal Medicine, The Ohio State University College of Medicine and Public Health, Columbus, OH, USA; ^3^Department of Pharmacology, The Ohio State University College of Medicine and Public Health, Columbus, OH, USA

**Keywords:** lung cancer, Fanconi anemia, pathway dysfunction, therapeutic target, FATSI

## Abstract

The Fanconi anemia (FA) pathway is a major mechanism of homologous recombination DNA repair. The functional readout of the pathway is activation through mono-ubiquitination of FANCD2 leading to nuclear foci of repair. We have recently developed an FA triple-staining immunofluorescence based method (FATSI) to evaluate FANCD2 foci formation in formalin fixed paraffin-embedded (FFPE) tumor samples. DNA-repair deficiencies have been considered of interest in lung cancer prevention, given the persistence of damage produced by cigarette smoke in this setting, as well as in treatment, given potential increased efficacy of DNA-damaging drugs. We screened 139 non-small cell lung cancer (NSCLC) FFPE tumors for FANCD2 foci formation by FATSI analysis. Among 104 evaluable tumors, 23 (22%) were FANCD2 foci negative, thus repair deficient. To evaluate and compare novel-targeted agents in the background of FA deficiency, we utilized RNAi technology to render several lung cancer cell lines FANCD2 deficient. Successful FANCD2 knockdown was confirmed by reduction in the FANCD2 protein. Subsequently, we treated the FA defective H1299D2-down and A549D2-down NSCLC cells and their FA competent counterparts (empty vector controls) with the PARP inhibitors veliparib (ABT-888) (5 μM) and BMN673 (0.5 μM), as well as the CHK1 inhibitor Arry-575 at a dose of 0.5 μM. We also treated the FA defective small cell lung cancer cell lines H719D2-down and H792D2-down and their controls with the BCL-2/XL inhibitor ABT-263 at a dose of 2 μM. The treated cells were harvested at 24, 48, and 72 h post treatment. MTT cell viability analysis showed that each agent was more cytotoxic to the FANCD2 knock-down cells. In all tests, the FA defective lung cancer cells had less viable cells as comparing to controls 72 h post treatment. Both MTT and clonogenic analyses comparing the two PARP inhibitors, showed that BMN673 was more potent compared to veliparib. Given that FA pathway plays essential roles in response to DNA damage, our results suggest that a subset of lung cancer patients are likely to be more susceptible to DNA cross-link based therapy, or to treatments in which additional repair mechanisms are targeted. These subjects can be identified through FATSI analysis. Clinical trials to evaluate this therapeutic concept are needed.

## Introduction

With more than 159,480 deaths estimated in 2013, lung cancer is the number one cancer killer in the United States (1). The standard first-line treatment of advanced lung cancer is platinum-based chemotherapy. However, response rates to chemotherapy vary widely among patients with the most common type, non-small cell lung cancer (NSCLC), likely due to heterogeneity in terms of platinum-sensitivity. Great efforts have been made to try to identify molecular predictive markers of platinum resistance. Inability to repair platinum adducts by the lack of nucleotide excision repair proteins (ERCC) has received considerable attention, as a potential predictor of the efficacy of adjuvant platinum-based chemotherapy. Results for this strategy, however, are conflicting (2, 3), possibly due to poor discrimination by antibodies of pertinent proteins isoforms.

Another major mechanism of DNA repair, related to homologous recombination, is through the Fanconi anemia (FA) pathway. FA genes collaborate to form foci of DNA repair on chromatin following DNA damage or during S phase of cell cycle (4). Cells with FA deficiency are hypersensitive to DNA damage agents such as cisplatin and mitomycin C (MMC) (4), and tumors from patients with germ line deficiency in some of the genes of this pathway have been shown to be sensitive to DNA-damaging agents, as well as inhibitors of other repair pathways, such as PARP inhibitors (4–6).

Additional studies have shown disruption of the FA cascade in sporadic cancers (7–9). These disruptions may involve epigenetic silencing of the FA-core complex, or mutations of one of several FA genes. The FA pathway contains 16 complementation groups, referred to as FA subtypes A, B, C, D1/BRCA2, D2, E, F, G, I, J, L, M, N, O, P, and Q. Eight of these proteins (A, B, C, E, F, G, L, and M) are subunits of FA-core complex 1, a nuclear E3 ubiquitin ligase (10–18).

The FA complex I functions to activate FANCD2 and FANCI by mono-ubiquitinating the protein following response to DNA damage (12, 13). The activated FANCD2 and FANCI proteins are subsequently transported to subnuclear foci, which are thought to be the sites of DNA repair and also contain BRCA1, FANCD1/BRCA2, proliferating cell nuclear antigen (PCNA) and Rad51 (12, 15, 19).

Given that the FA pathway plays an essential role in response to therapy-induced DNA interstrand cross-links, it is very plausible that cancers with defective FA pathway are more sensitive to cross-link based therapy. Since FANCD2 foci formation is critical for cancer cells to resist MMC and cisplatin, the best way to assess the functionality of this repair pathway as a whole is by evaluating FANCD2 foci formation. We have developed an FA triple-staining immunofluorescence based method (FATSI) to evaluate FANCD2 foci formation, and have generated preliminary data showing somatic deficiency of this pathway in tumors across several organ sites (20).

Herein, we report our evaluation of FA deficiency in a series of tumors from patients with NSCLC and the response of lung cancer cells with reduced FANCD2 expression (FANCD2 knock-down cell) to treatment with inhibitors of PARP, CHK1, and BCL-2/XL.

## Materials and Methods

### FA triple-staining immunofluorescence analysis

Human NSCLC samples were obtained from The Tissue Procurement Shared Resource of the Ohio State University (OSU) Comprehensive Cancer Center and The Cooperative Human Tissue Network, Midwestern Division at OSU, after Institutional Review Board (IRB) approval. FFPE tumor tissue was cut at 4 μm, placed on positively charged slides and stained with hematoxylin and eosin. Additional sections for immunofluorescence staining were placed in a 60°C oven for 1 h, cooled, deparaffinized, and rehydrated through xylenes and graded ethanol solutions to water in standard fashion. After antigen retrieval, the tissue sections were incubated with a primary antibody cocktail of rabbit polyclonal FANCD2 antibody (Novus Biologicals, Littleton, CO, USA) at a dilution of 1:1000 and a monoclonal anti-Ki67 mouse antibody (Dako, Carpenteria, CA, USA) at a dilution of 1:150, for 1 h at room temperature. Sections were then co-incubated with a secondary antibody (FITC conjugated to anti-rabbit IgG and Alexa fluor 594 donkey anti-mouse IgG, Invitrogen, Carlsbad, CA, USA) at 1:1000 for 1 h at room temperature. All rinses were performed on the autostainer with TBS-T. The sections were mounted on glass slides using a 4′ 6-diamidino-2-phenylindole (DAPI)-containing embedding medium (Vysis Dapi 1, Abbott Laboratories, Downers Grove, IL, USA). Formalin fixed paraffin-embedded (FFPE) FANCD2 foci negative cells (PD20) and foci positive cells (MCF-7 or FA corrected PD20) were used as controls on the sample slide during the procedure. The slides were analyzed under a 100× oil objective with a Nikon E-400 fluorescence microscope. See prior publication (20).

### Generation of FANCD2 knock-down cells

Lung cancer cells A549, H1299 (NSCLC) H719, and H792 (small-cell) were plated 24 h before transduction. At 60% confluence, cells were transduced with FANCD2-specific shRNA-expressing and puromycin-resistant lentiviral particles or control shRNA lentiviral particles (Santa Cruz Biotechnology Inc.) according to the manufacturer’s protocol. One day after incubation in medium containing polybrene agent, these transduced cells were transferred to a dish that contains normal growth medium. The transduced cells were selected in 4 mg/ml puromycin. To create stably transduced cells, 100–200 transduced cells were cultured in a 100 mm dish, and medium was replaced with fresh puromycin-containing medium every 3 days, until resistant colonies were identified. Twenty colonies were picked for each cell line, and then the colonies were expanded. Successful FANCD2 knockdown was confirmed by western blot. Veliparib, ABT-263, and BMN673 were obtained from Selleck Chemicals LLC; Arry-575 was provided by Array BioPharma.

### Cell viability analysis

Five thousand FA defective and control lung cancer cells from each line (H1299E/H1299D2-down, A549E/A549D2-down, H719E/H719D2-down, and H792E/H792D2-down) were seeded in each well of a 96-well plate 24 h prior to treatment. Cells were treated with the single agent at the designated dose (see Results). Dimethylthiazolyl-2-5-diphenyltetrazolium bromide (MTT) dye solution (Sigma, St. Louis, MO, USA) was added into the 96-well plate 20 h post treatment. The plate was incubated at 37°C for 4 h, and the treatment terminated by adding stop solution (isopropanol with 0.04 N HCl). MTT was cleaved by live cells to a colored formazan product. Absorbance at 560 nm wavelength was recorded using a Bio-Rad micro plate reader 680 (Bio-Rad Laboratories, Inc., Hercules, CA, USA). Each treatment was repeated in quadruplicate. An averaged absorbance of blank values (containing all reagents except cells) was subtracted from all absorbance to yield corrected absorbance. The relative absorbance of each sample was calculated by comparing the average of corrected absorbance with an average of corrected untreated control.

### Western immunoblot analysis and antibodies

Western immunoblot analysis was performed as described previously (21). Briefly, cells were digested with lysis buffer, which contained 250 mM NaCl; 5 mM EDTA; 1% Igepal; 5 mM dithiothreitol (DTT); 1 mM phenylmethylsulfonyl fluoride (PMSF); and 1% protease inhibitor cocktail (Sigma, Saint Louis, MO, USA). Protein concentrations were evaluated using the Bradford reagent (Bio-Rad, Hercules, CA, USA). Hundred micrograms of total protein was loaded onto NuPAGETM 4–12% Bis–Tris Gel (Invitrogen, Carlsbad, CA, USA). Protein on the gels was electro-transferred onto nitrocellulose membranes and blocked with blocking buffer (5% of non-fat milk, 500 mM of NaCl, 20 mM Tris, and 0.1% Tween-20). The membranes were incubated with primary antibodies at 4°C overnight. After washing with TBS-T (blocking buffer without milk) five times, 10 min each, the membranes were incubated with anti-mouse Ig or anti-rabbit Ig horseradish peroxidase linked to whole secondary antibodies (Amersham Pharmacia Biotech, Piscataway, NJ, USA) at room temperature for 1 h. After washing five times, 10 min each, a chemiluminescent detection system (ECL western blotting detection reagents, GE) was used to detect the secondary antibody. Finally, the membranes were exposed to x-ray films. Antibodies used were: rabbit polyclonal FANCD2 antibody (Novus Biologicals, Littleton, CO, USA), anti-tubulin monoclonal (Sigma, St. Louis, MI, USA).

## Results

### Fanconi Anemia pathway deficiency in non-small cell lung cancer tumor samples

**Figure 1:**
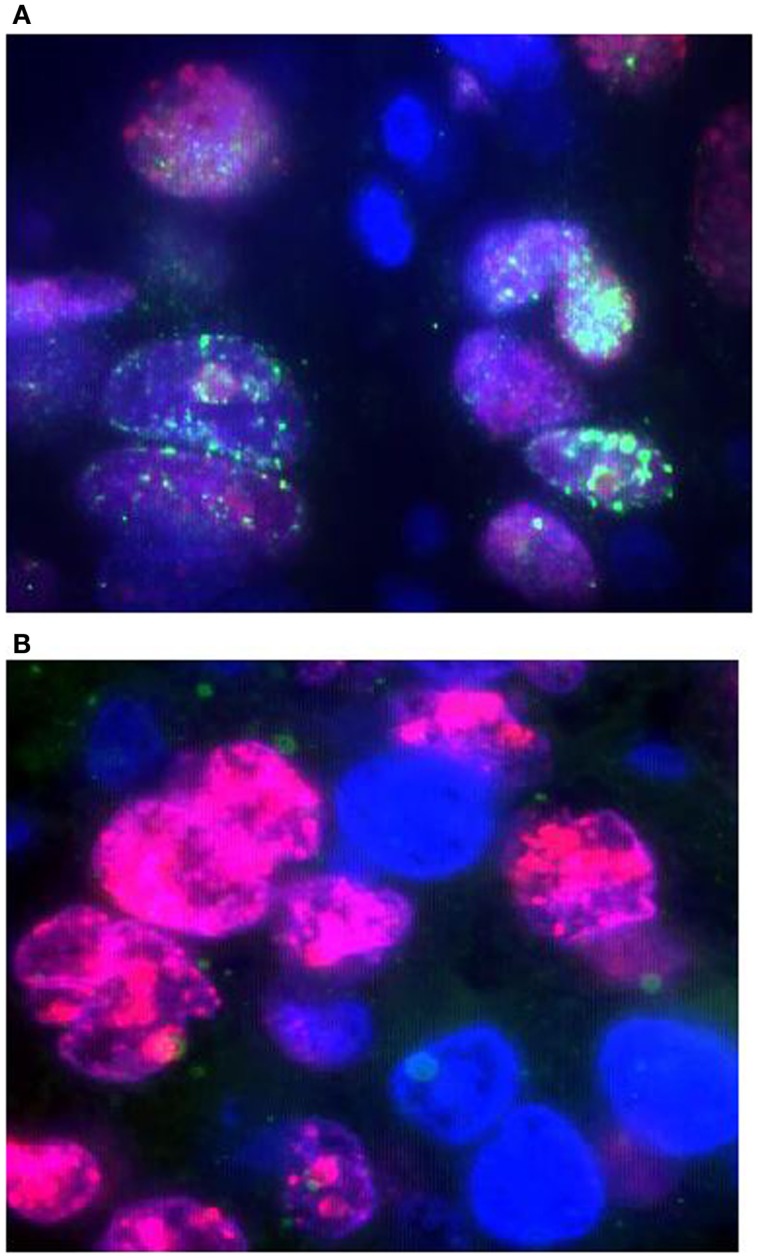
**Detection of FANCD2 foci formation in human lung tumors by the FATSI staining analysis**. The paraffin-embedded lung tumor tissues sections were deparaffinized and rehydrated. The tissue sections were incubated with a primary antibody cocktail of rabbit polyclonal FANCD2 antibody (Novus Biologicals, Littleton, CO, USA) at a dilution of 1:1000 and a monoclonal anti-Ki67 mouse antibody (Dako, Carpenteria, CA, USA) at a dilution of 1:150 for 1 h at room temperature. Sections then were incubated with a secondary antibody cocktail containing FITC conjugated anti-rabbit IgG and Alexafluor 594 donkey anti-mouse secondary for 1 h at room temperature. The sections were mounted on glass slides using a 4′ 6-diamidino-2-phenylindole (DAPI)-containing embedding medium (Vysis Dapi 1, Abbott Laboratories, Downers Grove, IL, USA). The slides were analyzed under a fluorescence microscope. **(A)** FANCD2 foci positive NSCL tumor, and **(B)** FANCD2 foci negative NSCL tumor. Magnification: 1000×.

We used the FATSI method to evaluate FANCD2 foci formation or lack thereof in lung cancer samples. We screened a total of 139 NSCLC FFPE tumors; 104 were evaluable for FANCD2 foci status (Figure 1). Eighty-one of the 104 (78%) evaluable tumors were found FANCD2 foci positive and 23 (22%) were foci negative.

Forty-nine of the NSCLC samples were of adenocarcinoma histology by morphology examination, 46 were squamous, 5 large cell, and 4 of mixed histology. Thirteen (26.5%) adenocarcinomas and seven (15.2%) squamous cell were foci negative. Two of the five large cell carcinoma and one of four mixed histology were foci negative. The frequencies may suggest that adenocarcinomas tumors have higher percentage of FANCD2 foci negative tumors as comparing to squamous cell carcinoma tumors. This observation will need corroboration with larger sample size and adjustment with other confirmatory tests, such as immunohistochemistry, not available to us for this dataset.

### Generation of FANCD2 knock-down cells and evaluation of sensitivity to PARP inhibitors

**Figure 2:**
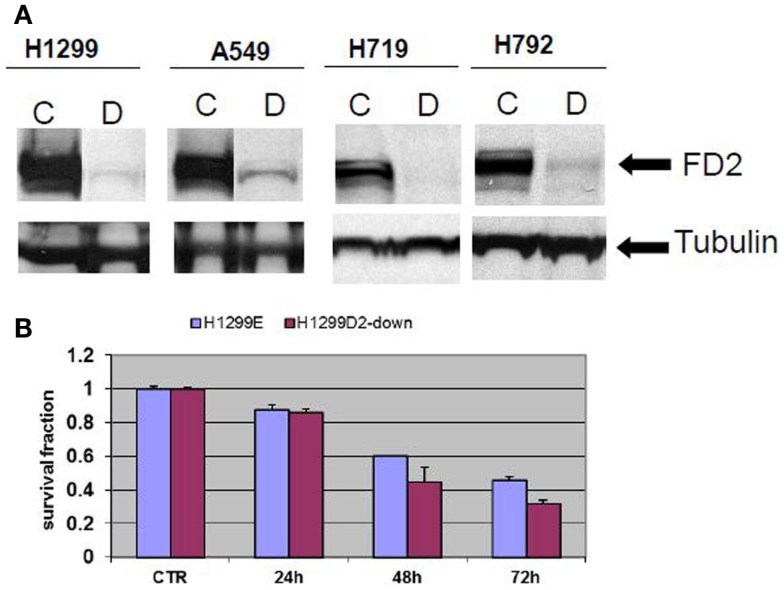
**Creating FANCD2 knock-down cells and evaluating response to cisplatin**. **(A)** NSCLC cells H1299, A549, and small cell lung cancer cells H719, H792 were plated. At 60% confluence, cells were transduced with FANCD2-specific shRNA-expressing and puromycin-resistant lentiviral particles or control shRNA lentiviral particles (Santa Cruz Biotechnology Inc.) according to the manufacturer’s protocol. The transduced cells were selected in 4 mg/ml puromycin to create stably transduced cells with reduced FANCD2 expression. Successful FANCD2 knockdown was confirmed by western blot detection of the FANCD2 protein. C is a control cell and D is FANCD2 knock-down cell. **(B)** The H1299E (H1299 was transfected with empty vector) and FANCD2 knock-down (H1299D2-down) lung cancer cells were treated with cisplatin (5 μg/ml) for 24, 48, and 72 h. The knock-down cell was more sensitive to the treatment.

Non-small cell lung cancer cell lines A549, H1299, and small cell H719, H792 were transduced with FANCD2-specific shRNA-expressing and puromycin-resistant lentiviral particles, or control shRNA lentiviral particles. To generate stably transduced cells, cells were selected by puromycin. Successful FANCD2 knock-down colonies were confirmed by western blot assessment of FANCD2 protein. Figure 2A illustrates four lung cancer cell lines with reduced FANCD2 protein. We also evaluated the response of the H1299E (H1299 cell transduced with empty vector) and FANCD2 knockdown (H1299D2-down) to treatment with cisplatin at a dose of 5 μg/ml, 72 h post treatment. We found that FANCD2 silencing resulted in sensitization of cells to cisplatin (Figure 2B).

**Figure 3:**
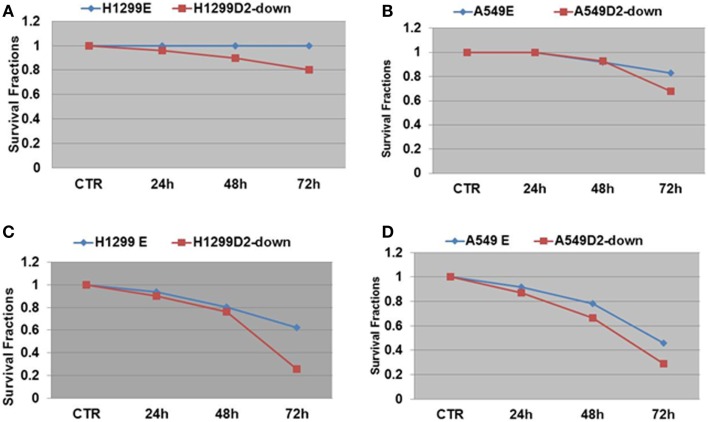
**MTT assay analysis of cell survival in FA-deficient and competent NSCLC cells post treatment with the PARP inhibitors veliparib and BMN673**. We treated the FA defective and control lung cancer cell lines H1299D2-down/H1299E and A549D2-down/A549E with veliparib (5 μM) or BMN673 (0.5 μM). MTT assay was used for the cell viability analysis and an averaged absorbance was recorded 24, 48, and 72 h post treatment. Cell viability analysis showed both control cells H1299E and A549E had no or limited response, and the FA defective cells H1299D2-down and A549D2-down had a mild response to the treatment of veliparib **(A,B)**. BMN673 had more cytotoxicity **(C,D)**.

To evaluate the influence of defective FA pathway in regards to cell viability following exposure to the PARP inhibitors veliparib (ABT-888) and BMN673, we treated the FA defective NSCLC cell lines H1299D2-down and A549D2-down, as well as their FA competent counterparts (H1299E and A549E) (empty vectors) with veliparib at a dose of 5 μM or BMN673 at dose of 0.5 μM. MTT assay was used for the cell viability analysis and an averaged absorbance was recorded 24, 48, and 72 h post treatment. Cell viability analysis showed that the FA defective H1299D2-down cells had 80% of viable cells compared to non-treatment controls 72 h post treatment with veliparib. In contrast, there was no influence on viability of the H1299E cell with the same treatment (Figure 3A). Both the A549D2-down cell and A549E cells responded to some degree to the treatment with veliparib at 48 and 72 h post treatment. The A549D2-down cells had 68% viable cells compared to 83% viable cells for the A549E cells, 72 h post treatment (Figure 3B).

BMN673 is a new class of PARP inhibitor, which has in addition strong PARP1-DNA complex trapping function (22, 23). Cell viability analysis showed that BMN673 was overall a more potent inhibitor (10-fold difference in active doses) compared to veliparib. H1299 FANCD2 knock-down cancer cells were also more sensitive to BMN673 compared to empty vectors transfected control cells (25 vs. 62% viable cells, respectively) 72 h post treatment (Figure 3C). A549D2-down cell had 29% viable cells and the A549E had 46% viable cells 72 h post treatment (Figure 3D). The IC50 of BMN673 treated A549E cell was 0.64 μM and the IC50 of A549D2-down was as low as 0.075 μM 72 h post treatment. The difference in the IC50 values between H1299E and H1299D2-down cells is smaller with 1.78 μM for the H1299E and 0.74 μM for the H1299D2-down.

**Figure 4:**
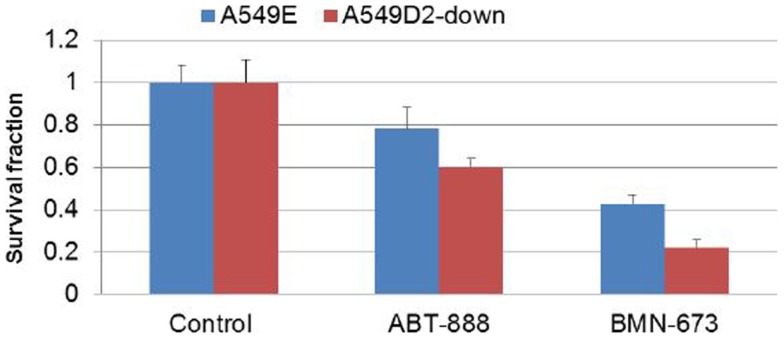
**Clonogenic analysis cell survival for A549E and A549D2-down cells treated with veliparib (0.5 μM) or BMN673 (0.5 μM): A549E and A549D2-down cells were seeded in a six-well plate and treated with or without veliparib (0.5 μM) or BMN673 (0.5 μM) for 8 days**. Colonies were stained with crystal violet and counted. Clonogenic survival analysis showed that veliparib alone was cytotoxic to A549 cells. The FA defective A549D2-down cells had 60% viable cells, and the A549E had 78% viable cells as compared to non-treatment control cells. Post treatment with BMN673 (0.5 μM), the FA defective A549D2-down cells has 22% viable cells, and the A549E had 43% viable cells as compared to non-treatment control.

To further investigate differential response to treatment with PARP veliparib and BMN673 between FA defective and FA intact lung cancer cells, we conducted clonogenic survival analysis. A549D2-down/A549E cells were seeded in a six-well plate and treated with veliparib (0.5 μM) or BMN673 (0.5 μM). Colonies were stained with crystal violet and counted. Clonogenic survival analysis showed that veliparib was cytotoxic to the FA defective A549D2-down cells (60% viable cells as compared to non-treatment control), and the A549E had 78% viable cells (Figure 4). Following treatment with BMN673 (0.5 μM), the FA defective A549D2-down cells were 22% viable as compared to non-treatment control. A549E cells were 43% viable (Figure 4).

### Effect of Fanconi anemia repair pathway integrity on response to checkpoint inhibitors

DNA-repair-deficient tumor cells have been shown to accumulate high levels of DNA damage. Therefore, the DNA-repair-deficient cells are dependent on other compensatory DNA-repair pathway, such as the CHK1-kinase pathway. FA defective cells are dependent on this G2/M checkpoint for viability, since the checkpoint activation allows for the repair of damaged DNA prior to mitosis. CHK1 is activated by the ATR kinase in response to DNA damage that stalls replication fork progression (24, 25). Defects in FA pathway have been shown to be synthetic lethal with CHK1 inhibition or genetic CHK1 depletion in human fibroblast and ovarian cancer cells (24).

**Figure 5:**
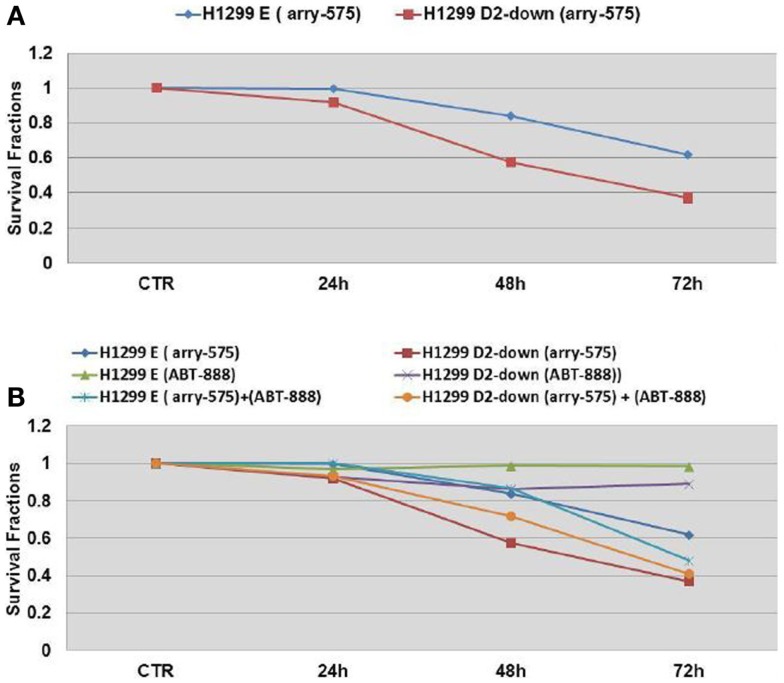
**Cell survival of FA defective lung cancer cells to treatment of CHK1 inhibitor as single agent or in combination with veliparib**. The FA defective lung cancer cell lines H1299D2-down and the control cell H1299E (transfected with empty vectors) were treated with Arry-575 at dose of 0.5 M. MTT assay was used for the cell viability analysis and an averaged absorbance was recorded 24, 48, and 72 h post treatment. Cell viability analysis showed the Arry-575 was more cytotoxic to these H1299D2-down cancer cells. The FA defective H1299 cells had 38% of viable cells compared to non-treatment controls 72 h post treatment. In contrast, there were 60% viable cells in the H1299E cells **(A)**. To evaluate the effect of the combination of PARP inhibitor and CHK1 inhibitor, we also treated the H1299D2-down/H1299E cells with Arry-575 (0.5 M) and veliparib (5 M) alone, or in combination for 72 h. A similar proportion of viable cells after treatment of Arry-575 alone and combination of Arry-575 and ATB888 was recorded **(B)**.

Arry-575 (GDC-0575) is a novel small molecule inhibitor of CHK1, in FA-deficient lung cancer cells. We conducted a dosage test on Arry-575 with H1299 cells, and found the IC50 values were around 1 and 0.5 μM for the H1299E and the H1299D2-down cells 72 h post treatment. We treated the FA defective lung cancer cell lines H1299D2-down and the control cell H1299E with Arry-575 at a dose of 0.5 μM. MTT assay was used for cell viability analysis and an averaged absorbance was recorded 24, 48, and 72 h post treatment. Cell viability analysis showed that Arry-575 was more cytotoxic to the H1299D2-down cancer cells. The FA defective H1299D2-down cells had 38% of viable cells compared to non-treatment controls 72 h post treatment. In contrast, there were about 60% viable cells in the control cell line H1299E cells (Figure 5A).

To evaluate potential synergy for the combination of PARP inhibition and CHK1 inhibition, we treated the H1299D2-down and the control cell H1299E with Arry-575 (0.5 μM) and veliparib (5 μM) alone, or in combination for 72 h. MTT assay analysis showed a similar portion of viable cell between the treatment of Arry-575 alone and the combination (Figure 5B).

### Response of FANCD2 defective small cell lung cancer cells to Bcl-2/Bcl-xL inhibition

Bcl-2 is a central apoptotic inhibitor, and overexpression is associated with tumor progression and treatment resistance in cancers. Overexpression has been reported in up to 80% of small cell lung cancers (SCLC). ABT-263 (navitoclax) is a potent and selective inhibitor of Bcl-2 and Bcl-xL, disrupting their interactions with pro-death proteins leading to the initiation of apoptosis (26, 27). However, a recent phase II study of single-agent navitoclax showed low rate of response to single-agent treatment in advanced and recurrent SCLC (28). Thus, pre-selection of patients most likely to derive benefit from BCL-2 inhibitors will be needed for further development of these agents in SCLC.

**Figure 6:**
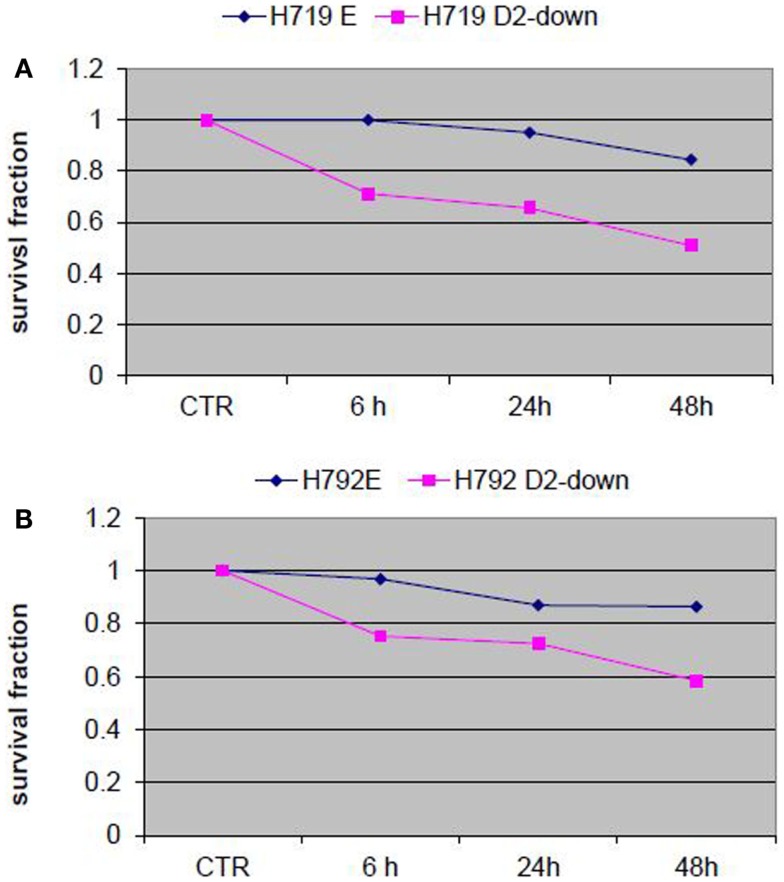
**Analysis of cell viability post treatment of small cell lung cancer cells with Bcl-2/Bcl-xL inhibitor ABT-263 (navitoclax)**. The FA defective small cell lung cancer cells H719D2-down and H792D2-down and their FA competent counterparts H719E and H792E were treated with navitoclax at a dose of 2 μM. The MTT assay was performed to determine cell viability 6, 24, and 48 h post treatment. Each treatment was repeated in quadruplets. An averaged absorbance of blank values (with no cells) was subtracted from all absorbance to yield corrected absorbance. The relative absorbance of each sample was calculated by comparing the average of corrected absorbance with an average of corrected untreated control. Each value presented in this figure was an average value obtained from four measurements. The cell viability analysis showed that navitoclax was more cytotoxic to both H719D2-down **(A)** and H792D2-down **(B)** FA defective cells.

To evaluate the influence of the FA pathway to treatment with navitoclax, the FA defective H719D2-down and H792D2-down cells as well as their FA competent counterparts (H719E and H792E) were treated with navitoclax at a dose of 2 μM. The treated cells were then harvested at 6, 24, and 48 h post treatment. MTT cell viability analysis showed that navitoclax was more cytotoxic to the FA-deficient H719D2-down compared to its control (51 and 85% viable cells at 48 h, respectively) (Figure 6A). Similarly, the H792D2-down small cell lung cancer cells had 58% viable cells and the H792E had 86% viable, 48 h post treatment (Figure 6B).

### Compensatory activation of alternative DNA-repair pathways following exposure to veliparib

**Figure 7:**
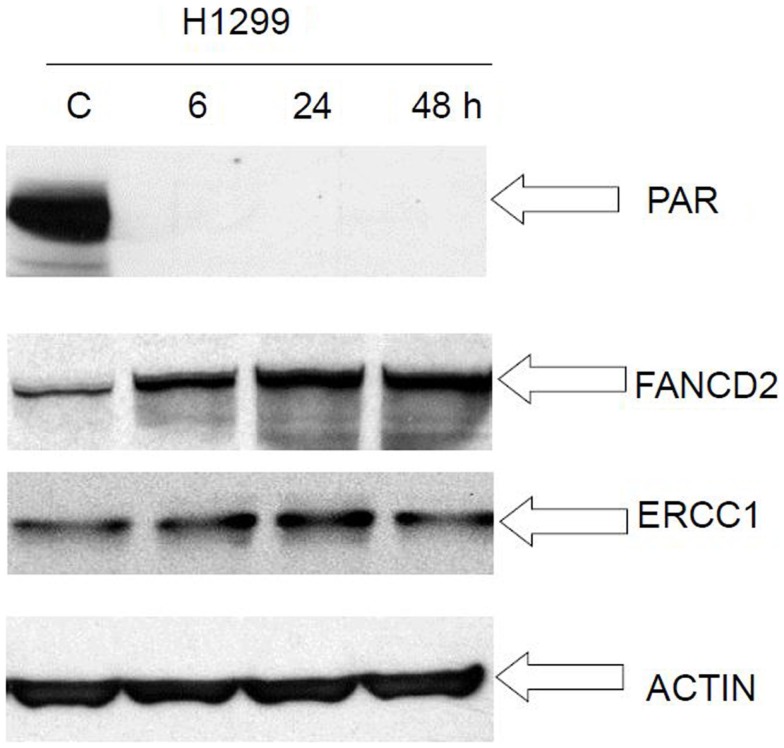
**Western blot analysis of FANCD2 and ERCC1 expression in lung cancer cell H1299 following treatment with veliparib**. PAR protein level was reduced at 6 h post veliparib 5 μM exposure and maintained at low levels through 48 h in the H1299 cells. However, FANCD2 and ERCC1 protein expression raised simultaneously in these cells post treatment with veliparib. The data suggest that veliparib induces compensatory activation of alternative DNA-repair pathways. C is control (without treatment).

We performed Western immunoblot analysis to evaluate the expression level of PAR, FancD2, and ERCC1, in the human cancer cell lines H1299 following exposure to veliparib. PAR protein level was reduced at 6 h post veliparib exposure (5 μM) and maintained at low levels through 48 h in the H1299 cells. However, FANCD2 and ERCC1 protein expression was simultaneously elevated in these cells post treatment with veliparib (Figure 7).

## Discussion

DNA repair is essential for cells to maintain genome stability. There is a growing appreciation that defects in homologous recombination repair underlie hereditary and sporadic tumorigenesis, conferring a survival advantage to cancer cells. However, this deficiency may increase sensitivity of tumors to certain DNA-damaging agents. Homologous recombination deficiency may therefore prove to be a target of cancer treatment, as long as appropriate biomarkers become available to identify patients with these tumors (29). Our recently developed FATSI method to evaluate FANCD2 foci formation, which is capable of evaluating the functionality of the pathway using FFPE tumor samples (20) could represent such a test. This method is suitable for large scale screening to select cancer patients most suitable for treatment with DNA-damaging agents. In addition, the therapeutic window for certain novel molecular targeted agents such as PARP inhibitors, checkpoint inhibitors, and BCL-2/xL inhibitors may be larger for the FA-deficient tumors because the DNA lesions induced cannot be efficiently repaired and will eventually lead to the cells undergoing apoptosis (29, 30).

We found that 22% of NSCLC tumors examined had functional deficiency in the FA pathway and that cells with deficient FA pathway were more sensitive to treatment with PARP inhibitors. Both MTT and clonogenic analyses showed BMN673 was more potent compared to veliparib. This may be due to veliparib having a much weaker ability to trap PARP–DNA complexes despite its great activity as a PARP catalytic inhibitor (23). Furthermore, our studies showed that lung cancer cells with deficient FA pathway were more sensitive to treatment with a CHK1-kinase pathway inhibitor and a BCL-2/XL inhibitor.

Of concern are the results showing that veliparib up-regulated FA and nucleotide excision repair proteins in the H1299 FA wild type cells. Thus, treatment with veliparib in repair wild type cells may plausibly influence resistance to DNA targeting cytotoxic chemotherapy. Cancers with defective FA or NER pathways, which are incapable of mounting a compensatory response, may represent a better target for veliparib (or BMN673) alone or in combination with DNA targeted cytotoxic chemotherapy. It is unclear at this point if targeting two additional repair mechanisms in the setting of FA dysfunction will be better than one in tumor shrinkage and/or delaying the appearance of resistance. That is, for example, inhibiting base excision repair through PARP inhibition/PARP trapping and nucleotide excision repair through ERCC1 in FA-deficient tumors. It is possible that the risks of additional toxicities may outweigh any potential benefits. However, these experiments are worth conducting but it would be optimal to use best in class drugs.

The identification of patients with somatic functional deficiency of the FA pathway in their tumors may also lead to a better understanding of the specific genetic/epigenetic events that drive the cancer in these patients, by selecting these patients for deep DNA and RNA sequencing and methylome analysis. The genetic instability caused by the repair deficiency may lead to additional molecular changes that take over as drivers, a concept that have been named non-oncogenic addiction or induced sustainability (31, 32). Identification and inactivation of these added drivers may result in an opportunity for synthetic lethality. Our laboratory is pursuing this approach by performing RNAseq in FA-deficient tumor archival material and in fresh biopsies from patients in clinical trials of PARP inhibition.

Resistance to DNA interactive agents and PARP inhibitor may develop in patients with FA dysfunctional tumors through the recovering of function after a period of treatment. It has been reported that promoter methylation of several FA genes resulted in deficiency in FA repair foci formation in human cancers (4, 33–38). The plasticity of epigenetics changes may lead, for example, to hypomethylation of these promoters with a resulting recovery of FA function. Other reported mechanisms of function recovery include post-treatment restorative acquired mutations in previously dysfunctional repair genes (39, 40). Thus, it is of tantamount importance that any patient selection for clinical trials evaluating therapeutics in FA-deficient tumors is based on screening of recent tumor material, not separated by intervening treatment. Requiring biopsies once progression occurs will offer invaluable information regarding the mechanisms mediating acquired resistance.

In summary, the FATSI method shows that a proportion of lung cancer patients have tumors with FA deficiency. We have also demonstrated that lung cancer cells with defective FA pathway were more sensitive to PARP inhibitors and increase the therapeutic window of other molecularly targeted agents. Clinical studies are needed to validate the therapeutic potential of these preclinical findings.

## Conflict of Interest Statement

The authors declare that the research was conducted in the absence of any commercial or financial relationships that could be construed as a potential conflict of interest.
